# Marangoni effect induced macro porous surface films prepared through a facile sol-gel route

**DOI:** 10.1038/s41598-017-05506-7

**Published:** 2017-07-13

**Authors:** Shahid Khan, Kangkai Wang, Guangzhong Yuan, Mahmood ul Haq, Zhizheng Wu, Muhammad Usman, Chenlu Song, Gaorong Han, Yong Liu

**Affiliations:** 1 State Key Laboratory of Silicon Materials and School of Materials Science and Engineering, Zhejiang University, Hangzhou, 310027 China; 2 Key Laboratory of Advanced Materials and Applications for Batteries of Zhejiang Province, Zhejiang Province, China; 3 Experimental Physics Laboratories, National Centre for Physics, Shahdara Valley Road, Quaid-i-Azam University, Islamabad, Pakistan

## Abstract

Based on TiO_2_ as a model system, the sol-gel one step facile method is established to fabricate the macro-porous morphology films on the basis of Marangoni effect. In this proposed mechanism, the binary mixture of hydrophilic CuCl_2_ and lipophilic Ti-O network is used in *sol* to produce phase separation. A suitable evaporation rate in the *gel* film process leads to the macro-porous film due to Marangoni effect. It is observed that the macro-porous morphology of the film sustains during the annealing process, which suggests the creation of porous surface morphology in *gel* film stage rather than due to annealing. To analyze the preparation mechanism, the *sol-gel* process and microstructure of films are examined using TG-DTA, SEM, TEM, XRD, Raman, UV-Vis, XPS and FTIR. Furthermore, the optical-thermal properties are studied for the potential applications of such porous surface films as solar selective absorber.

## Introduction

Porous materials are getting great interest in various fields, Nakanishi and Soga (1991)^1^ first reported the synthesis of macro-porous silica monoliths. After that a lot of efforts have been made to develop the synthesis of porous materials. Generally, a substance with controlled pore structure in terms of pore size distribution, porosity and shape is very useful in different applications such as electronics^2, 3^, energy storage^4^, catalysis^5^, sensing^6^, and biomedical sciences^7^ etc.

Porous materials have exceptional characteristics with particular significance of high accessible surface area, and low density. In particular, porous TiO_2_ thin films with large specific surface area are predictable to enclose a wide range of applications for instance, optical cells, solar energy conversion, and high efficient catalysts. Porous surface morphology generating by phase separation, phase separation generates porous surface morphology is consider to be an advance technology to produce product having many application which other method could not achieve, has become an efficient method for formation of new TiO_2_ porous thin film. By using proper process parameter and selection of system, various morphology and hierarchical pores materials could be achieve, which lead to the production of functional materials^8^. The porous TiO_2_ had attracted enormous interests and advantages in the past decades but TiO_2_ has a wide band gap of 3.2 eV which is a drawback to absorb visible light, only absorb UV light which include only 4% of solar spectrum. Hence, its optical response is limited in that region. This limitation could be overcome by two promising ways *i.e*. texture surface and nano composite. Lots of research has been made on texture surface and nano composite to enhance visible light absorption. E.Barrera-Calva *et al*.^9^. reported the Silica-copper oxide (silica-CuO) composite thin films prepared by a dipping sol-gel route for solar selective absorber coatings. Sajid Ali Ansari *et al*.^10^. Studied the nano-composite of Au-TiO_2_ for visible light activity. They also reported the Ag@m-TiO_2_ nano-composite for high Photocatalyst and photo electro-chemical properties due to synergetic effect of surface plasmon resonance of Ag nano particle^11^. Porous TiO_2_ films have previously been prepared through various techniques such as hydrothermal crystallization^12^, direct deposition^13, 14^, sputtering technology^15^, ultrasonic spray pyrolysis^16^, and sol-gel method^17^. In sol-gel method, these were prepared in the presence of polyethylene glycol^18–20^, polyoxyethylene^21^, or nonylphenyl ether^22, 23^, etc. as surfactant and colloidal templates.

In our last paper^24^ we have focused on the effects of the Cu content upon the solar selective properties of the porous composite coatings. In this work, by study the annealing temperature effects upon the structure of the films, we try to address the mechanism of the synthesis through Marangoni effects.

Surface-tension-driven convection, well-known as Marangoni effect, is of great interest in the study of material processing, especially for 2-D materials with ordered patterns^25^. It has been known from early 19th century that the functioning of the Marangoni flow along the free liquid surface would be induced through a surface tension gradient, in contrary, surface tension gradient emerged through the composition or temperature variation being investigated^26^. However, the useful sol-gel one step method in the absence of any surfactant or template on the basis of Marangoni effect is not reported yet.

In the present work, we have tried to achieve a porous surface morphology based on TiO_2_ as a model system by a facile one step sol-gel method on the basis of Marangoni effect. The preparation mechanism of such porous morphology is discussed in details. Furthermore, the optical-thermal properties of such a complementary structural film have also been studied as a solar selective absorber (SSA). The porous surface morphology was designed as a light-trapping factor, which enhance the absorption through multi-reflections.

## Results and Discussion

**Figure 1:**
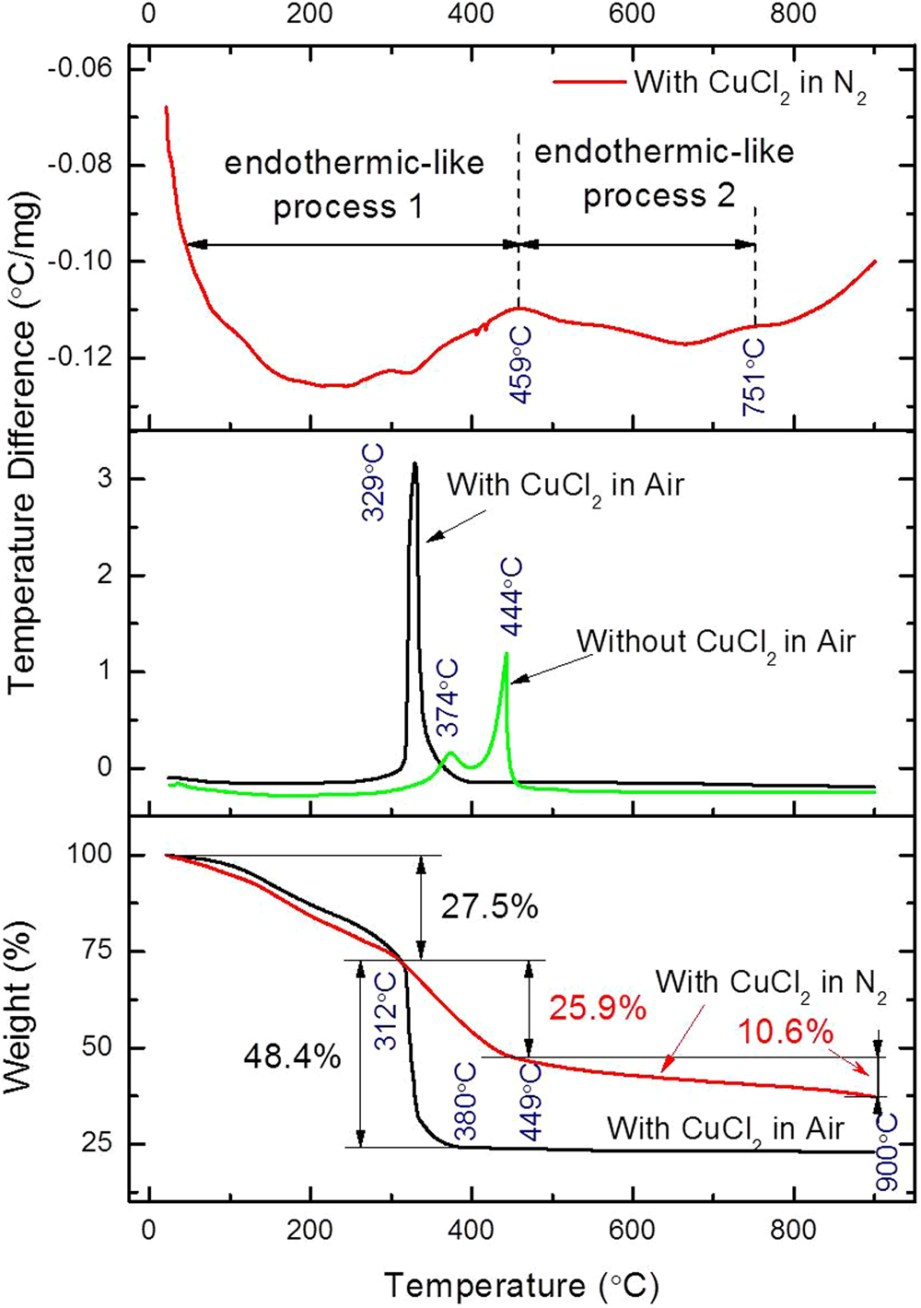
The TG-DTA curves of the xerogel in two atmospheres including air and flowing N_2_.

The results of TG-DTA in different ambient are shown in Fig. 1 to analyze the pyrolysis process. The upper two panels show the outcome of DTA with different ambient, the first one shows the xerogel annealed in N_2_ atmosphere while the second shows annealed in air with or without CuCl_2_ in the precursor.

In N_2_ atmosphere, *i.e*. an oxygen-free environment similar to the vacuum annealing condition, (we have only plotted the results of xerogel in the presence of CuCl_2_ because there is no significant difference in the results obtained without CuCl_2_) two wide and weak endothermic-like peaks are observed; featuring a typical pyrolysis process involved in the transformation from organics to carbon. On the other hand, the samples annealed in air, which contain CuCl_2_ precursor, only one intensive exothermic peak around 329 °C is seen. This peak corresponds to the combustion of carbon precursors, mainly attributed to SA. For the sample without CuCl_2_ precursor, two exothermic peaks are observed. The first peak located around 374 °C corresponds to the pre-combustion of some small organics without benzene rings. Whereas, the second exothermic peak around 444 °C is related to the combustion of SA, which has a more firm benzene structure rather than chain hydrocarbons. The combustion peak of carbon is generally beyond 400 °C. The peak shift indicates that Cu is more active and hence it could accelerate the combustion of carbon precursor in the air.

The last panel of Fig. 1 shows the TG curve in different conditions. In this analysis, the mass loss evolution could be explained as follows. In low temperature range (from room temperature to about 312 °C) two TG curves are almost the same, and the 27.5% weight loss is mainly attributed to the evaporation of some small and low boiling molecules. As the temperature increases to around 380 °C for the TG curve processed in air, a drastic drop of weight about 48.4% occurs, accompanied with the intensive exothermic peak in the DTA curve, which could be due to the combustion of organics. While for the TG curve processed in N_2_, two linear weight losses with different rates take place, *i.e*. in the temperature range 312 °C to 449 °C (25.9% loss) and 449 °C to 900 °C (10.6% loss), respectively. The former demonstrates a fast pyrolysis process from organics to carbon materials, while the latter corresponds to a more thorough carbonization involving the releasing of some dangling and light molecules like H. Thus, the carbonization process during the annealing treatment in the oxygen-free environment could be confirmed, and the annealing temperature of about 600 °C in vacuum is believed to be sufficient enough for the carbonization of the precursor.

**Figure 2:**
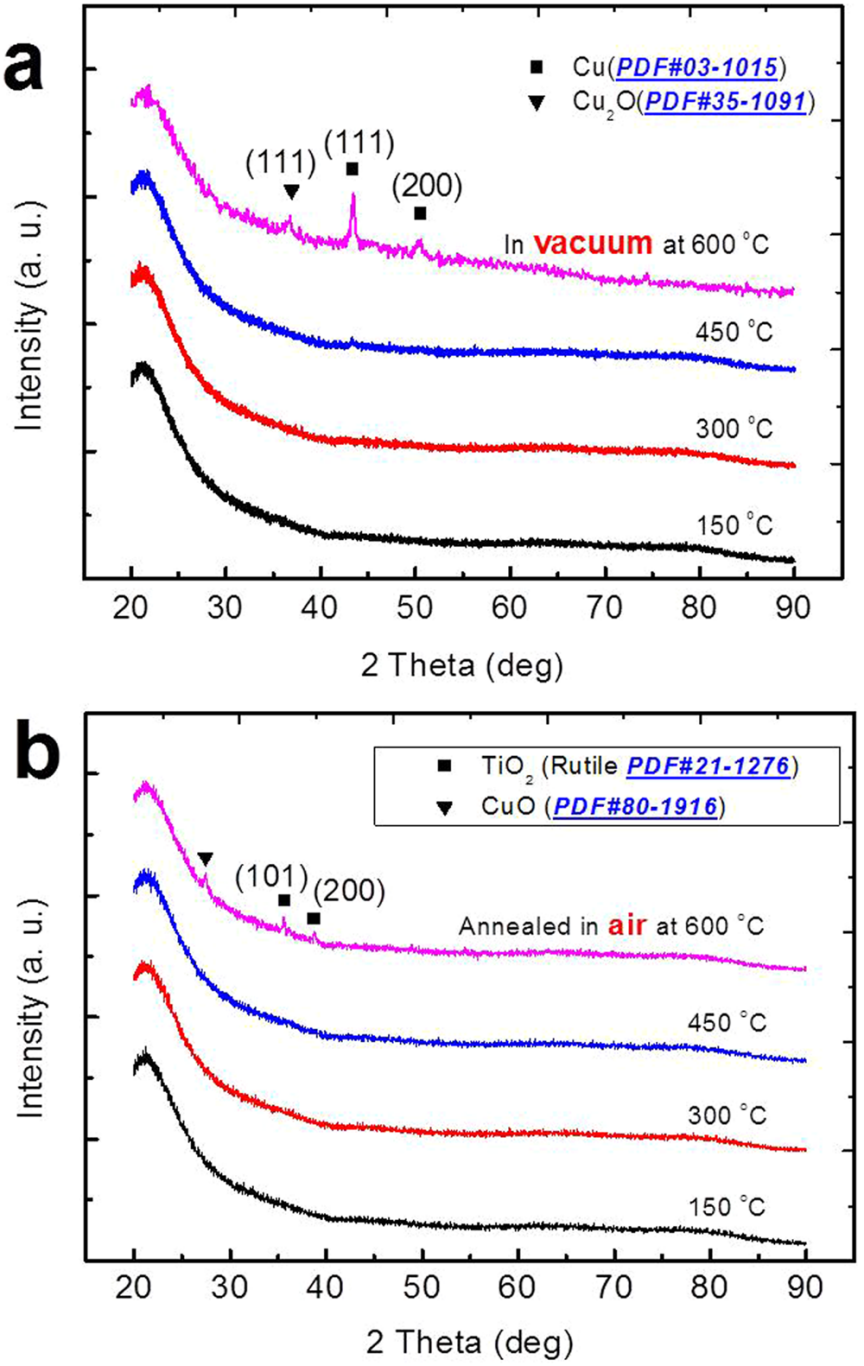
The XRD patterns of the samples with different Temperature in the range 150 to 600 °C (**a**) Vacuumed annealing (**b**) Air annealing.

Figure 2 shows XRD patterns of the films annealed in vacuum and air atmosphere at different temperatures *i.e*. (150, 300, 450, and 600) °C. The results of samples annealed in vacuum atmosphere are depicted in Fig. 2a. The samples annealed in the temperature range of 150–450 °C show no crystalline phase which could reflect a complete amorphous structure of the films. However, as the temperature increases to 600 °C three peaks are observed, which are assigned to Cu (111), (200) and Cu_2_O (111) cubic phase. The intensity of Cu peak is slightly higher as compared to Cu_2_O which may be due to the fact that CuCl_2_ is finally compacted to elementary Cu during the carbonization process. No crystalline phase of TiO_2_ was found due to the lack of oxygen as samples annealed in vacuum, the other reason maybe the steric effect in the pyrolysis process.

The XRD results of samples annealed in air atmosphere are shown in Fig. 2b in the temperature range of 150–450 °C, where no crystalline phase of the film is observed, similarly as in the case of vacuum annealing. But again as the temperature increases to 600 °C, low intensity peaks of crystalline rutile phase of TiO_2_ (101) appeared which is due to the fact that in air atmosphere carbon combustion provides a lot of heat which promotes direct generation of TiO_2_. The CuO peaks also appeared in this spectrum but no peak of Cu was observed, as the samples annealed in air so the organic burn out happened.

**Figure 3:**
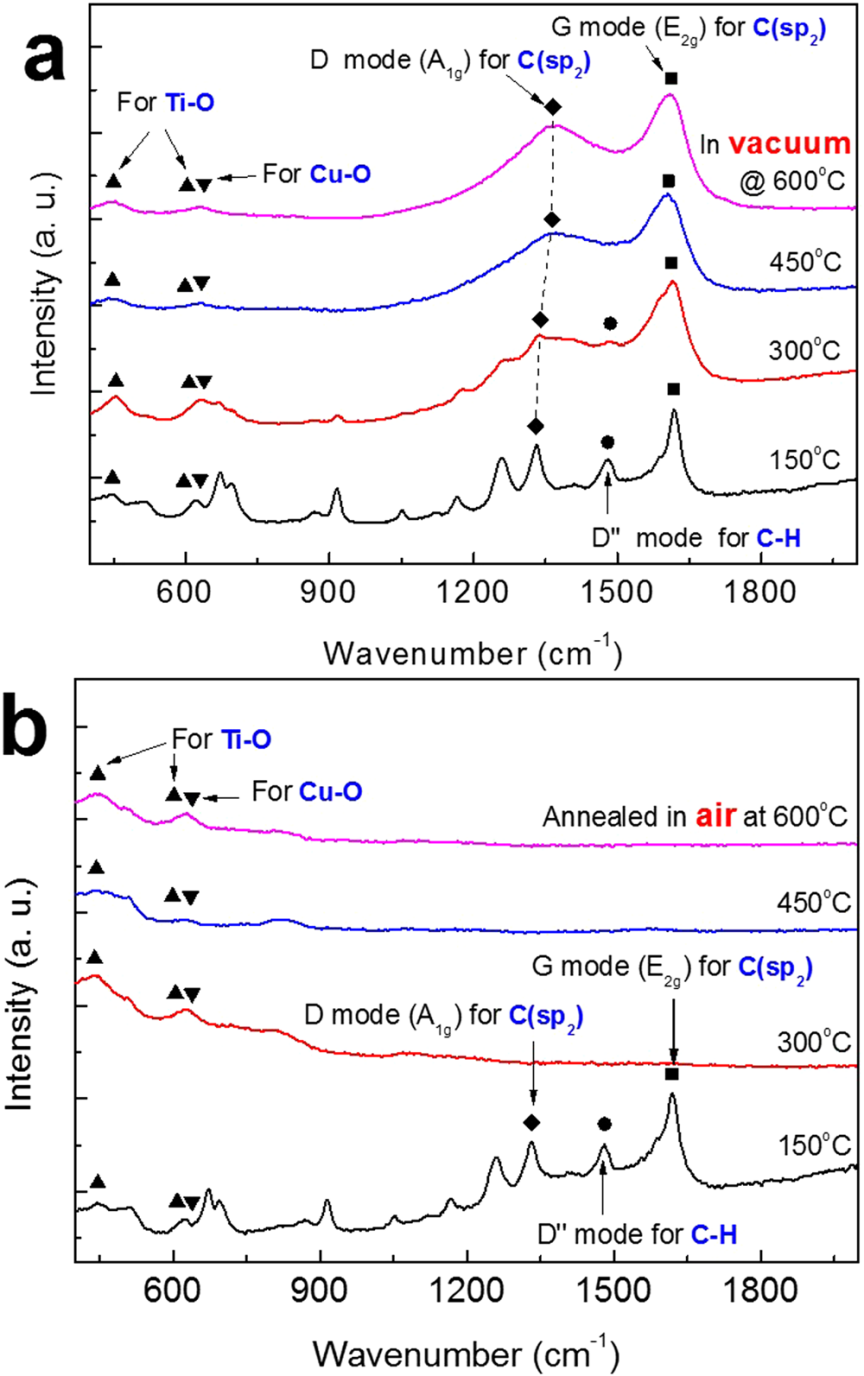
The Raman spectra of the samples with different Temperature in the range 150 to 600 °C (**a**) Vacuum annealing (**b**) Air annealing.

Figure 3(a and b) shows the Raman spectra of the samples annealed in Vacuum and Air atmosphere respectively. Figure 3a shows the Raman spectra of samples annealed in vacuum with different temperatures. At 150 °C several peaks of different organics were observed in the wavelength range from 700 to 1260 nm. Unfortunately, only few peaks could be recognized, such as the peak at 1000 cm^−1^ could be assigned to Diamond like carbon (DLC), but the peak at 1260 cm^−1^ is still controversial between nano diamond-like crystalline carbon (NDLC), *sp* ^3^, and *sp* ^2^ signals.

The prominent peaks assigned from Raman spectra are D mode (K-point phonons of *A*_1g_ symmetry), G peak (zone center phonons of *E*_2g_ symmetry) and some peaks due to Ti-O and Cu-O are also observed. But as the temperature increases from 300 to 450 °C most of the unassigned peaks disappear due to the removal of organics at higher temperature but the D and G peaks of Carbon are still present at the highest temperature of 600 °C. However, the peaks positions are slightly shifted towards higher wavelength. This trend suggests that the Carbon atoms are arranged in proper order.

Figure 3b shows the Raman spectra of the samples annealed in air atmosphere. At the lowest temperature of 150 °C the results are almost the same as in the case of vacuum annealing with multiple organics’ peaks; D and G peaks of carbon are also present in this plot. But at higher temperature range *i.e*. 300, 450, 600 °C, most of the peaks are destroyed including D and G peaks due to burning of organics in the presence of oxygen in air atmosphere leaving smaller peaks of Ti-O and Cu-O network. This satisfies XRD results of 600 °C annealed samples in air atmosphere where no Cu was found and only CuO was present there.

**Figure 4:**
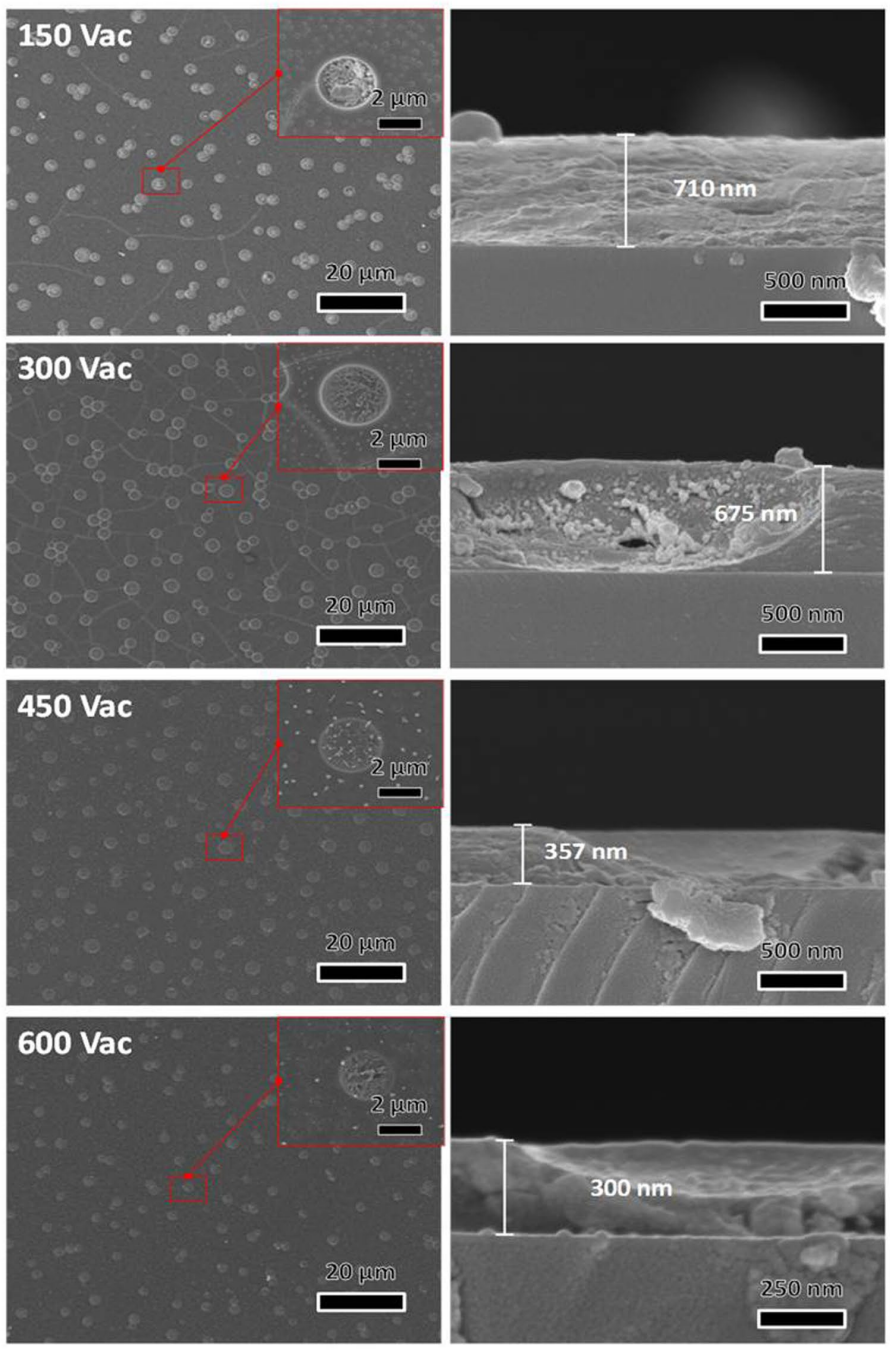
Surface and cross-sectional SEM images of samples annealed in Vacuum atmosphere with different temperature in the range 150 to 600 °C.

Figure 4 shows the surface and cross-sectional images of vacuum annealed samples. The surface morphology (left column) at the lowest temperature of 150 °C shows hierarchical porous morphology with small cracks on the surface. Similarly for 300 °C temperature the surface also exhibits small cracks which is due to the fact that at such low temperatures there is a large number of residual organic matter left and the thickness of samples is high (see right column). As the temperature increases to 450 and 600 °C, the cracks are removed and thickness is reduced. The inset picture of surface morphology revealed that the morphology changes inside the holes with annealing temperature which will be explain later.

The cross-sectional images of films reveal the thickness variation at different temperatures. At 150 °C the thickness of the samples was ~710 nm, which is due to large number of residual organics. As the temperature increases to 450 and 600 °C, the thickness of the films decreases to ~357 and ~300 nm respectively due to the decrease of residual organics, as it was expected from TG analysis.

**Figure 5:**
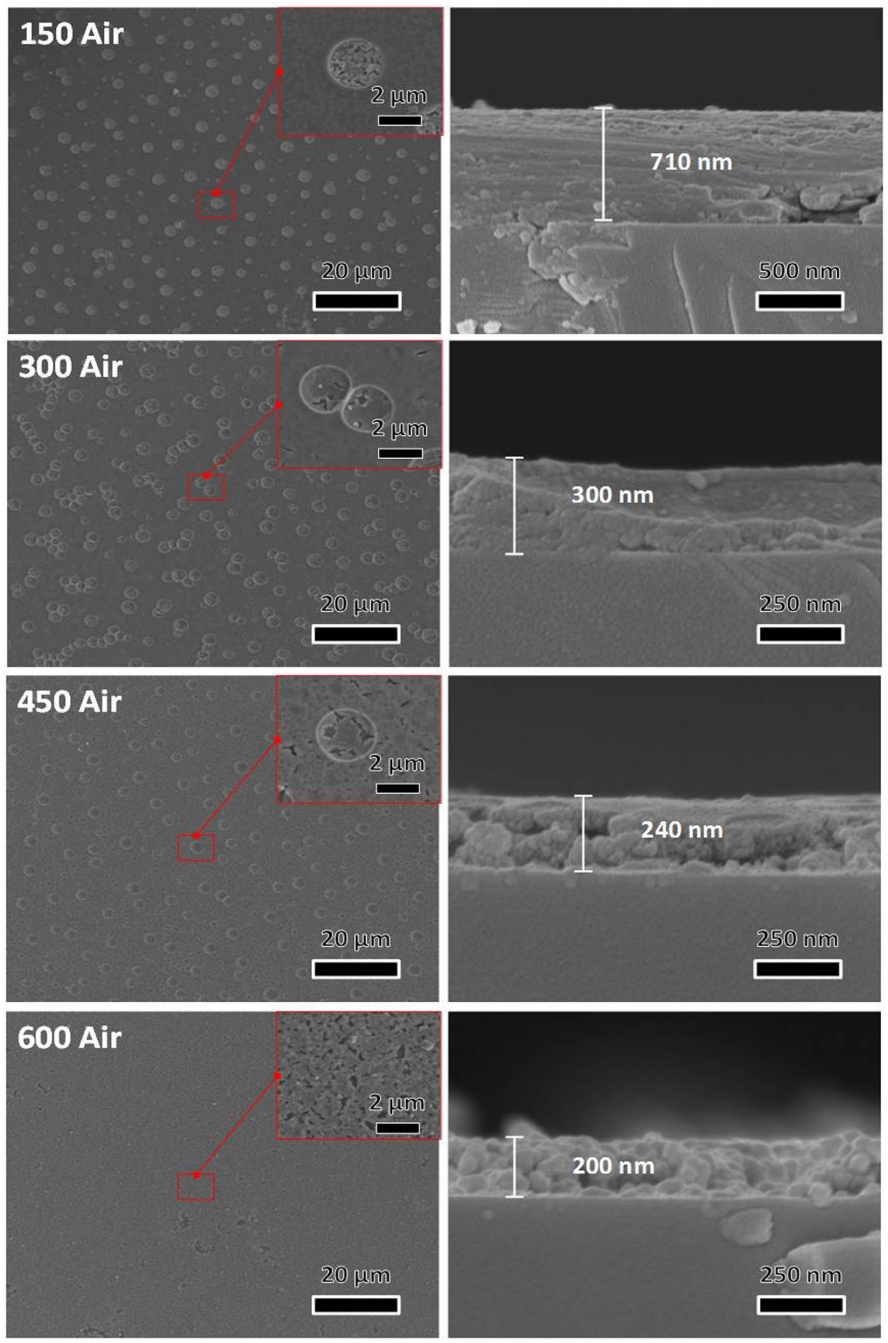
Surface and cross-sectional SEM images of samples annealed in Air atmosphere with different temperature in the range 150 to 600 °C.

Figure 5 shows the surface and cross-sectional images of air annealed samples. The sample annealed in 150 °C in the air almost shows the same surface and cross-section results as the vacuum annealed sample, except reduced number of cracks. This propose that low temperature in different annealing environment does not show a big difference in surface morphology, which confirms that the porous surface was constructed in the gel-film process. On the other hand, at higher temperatures of 300, 450 and 600 °C, a slightly different result was observed as compared to vacuum annealing. In this case, thickness of the samples decreases significantly to 300, 240, and 200 nm for respective temperatures. The surface morphology at higher temperatures such as 450 and 600 °C is also changed, and at 600 °C the surface is almost collapsed with no macro-pores appeared in the film. This proves the fact that all the organics are burnt out in the presence of oxygen.

**Figure 6:**
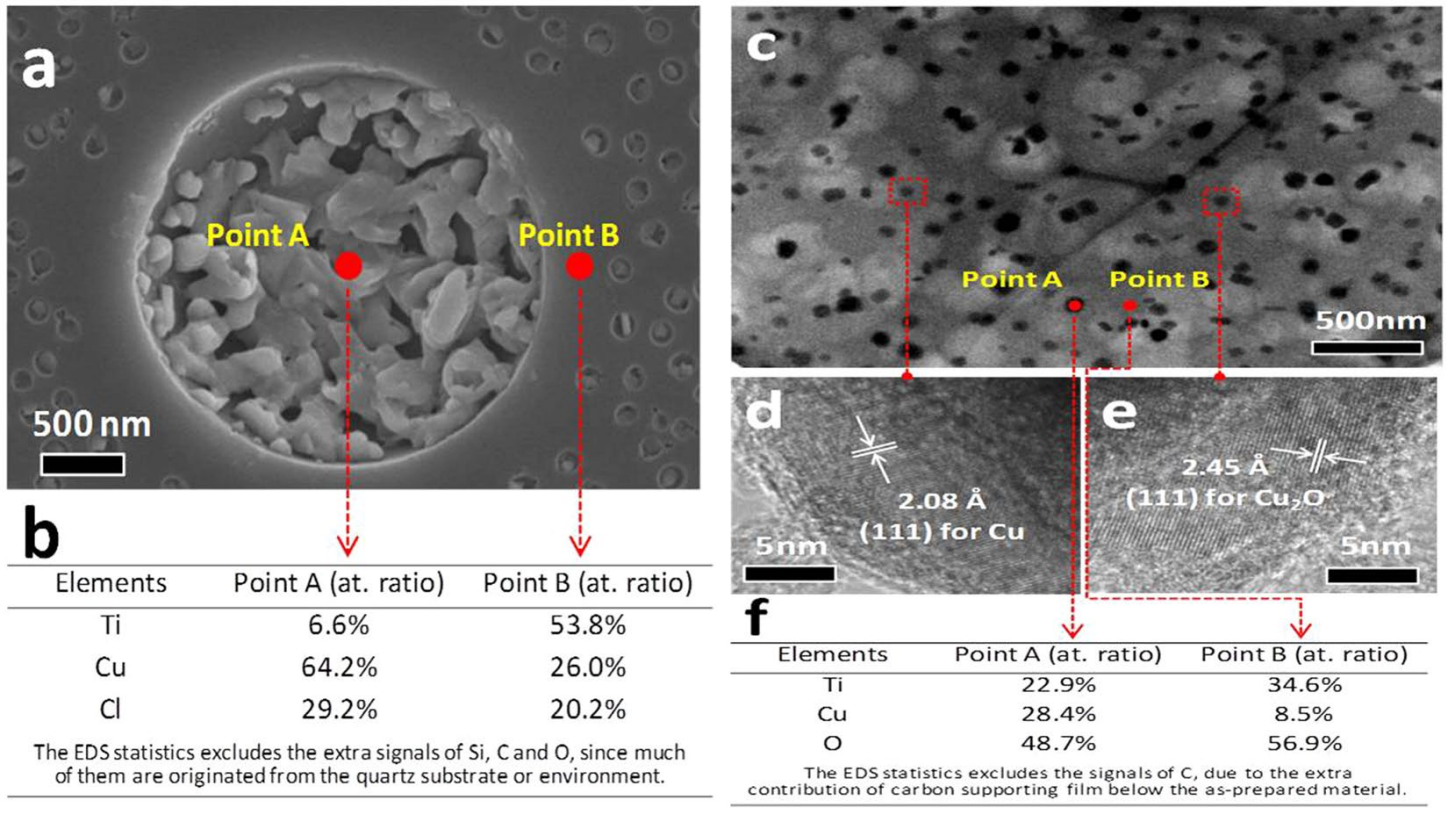
Microstructure and composition analysis (**a**) SEM image of the sample annealed at 150 °C in air (**b**) EDS (**c**) the overview TEM image of the sample annealed at 600 °C in vacuum (**d**,**e**) HRTEM (**f**) EDS.

Further microstructural and compositional analysis is shown in Fig. 6. The EDS results of two points highlighted in the SEM image (Fig. 6a) of the sample annealed at 150 °C in air indicate that at point A there is a large amount of Cu (about 64 a.t.% ratio) as compared to the Ti (about 6.6 a.t.%). This shows that CuCl_2_ precursor is successfully dissolved in ethanol and embedded inside the pore which is a Cu rich phase. While at point B there is a large amount of Ti is present with smaller amount of Cu (Cu poor phase), which provides phase separation. Note that the EDS results include some extra signals of Si, C and O which are not included here, since many of them are originated from quartz substrate or environment.

From the overview TEM image of the sample annealed at 600 °C in vacuum (Fig. 6c), it can be observed that the pores are spread throughout the film with few additional black spots in the figure. The high resolution TEM images, shown in Fig. 6(d and e), indicate that two black spots selected are assigned to two types of diffraction fringes: one belongs to the Cu rich region of (111) with the spacing of 2.08 Å and the other is assigned to Cu_2_O (111) with inter-planer spacing of 2.45 Å, which confirms that the CuCl_2_ precursor is converted to Cu and Cu_2_O.

Figure 6f shows EDS analyses of two selected points. Point A indicates the black spot in the film which is Cu rich region with a relatively high concentration ~28.4 a.t. % and the Point B correspond to the Cu poor region with low concentration ~8.5 a.t. %, which agree with SEM results (Fig. 6b).

**Figure 7:**
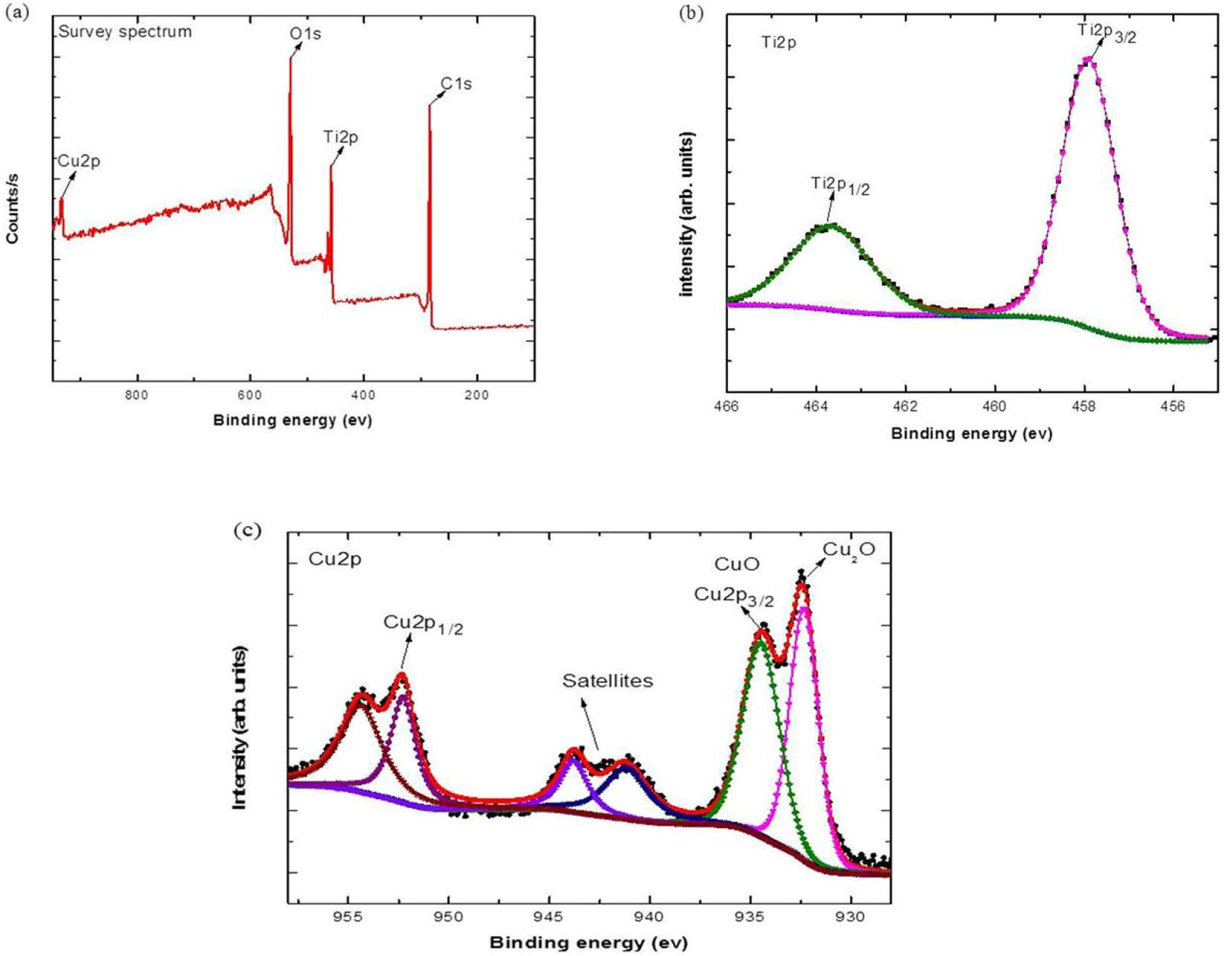
XPS spectrum (**a**) Full survey spectrum (**b**) High resolution fitted spectrum of Ti2p (**c**) High resolution fitted spectrum of Cu2p.

In order to analyze the chemical state of the as prepared films X-ray photoelectron spectroscopy (XPS) were carried out. Figure 7a show the full survey spectrum of as prepared samples that confirmed the presence of C1s, Ti2p, O1s and Cu2p without any impurities which, is in good agreement with XRD and EDX results. The high resolution fitted XPS spectra of Ti2p and Cu2p are displayed in Fig. 7(b and c). The Ti2p spectra show two individual peaks at (457.9 ± 0.02 and 463.9 ± 0.02) eV which corresponds to Ti2p_3/2_ and Ti2p_1/2_, respectively. The peak sitting between two peaks is *ca*. 5.8 suggesting the Ti^4+^ oxidation state exists. Figure 7c provides the high resolution fitted XPS spectra of Cu2p. It shows the oxidation states of Cu, one peak located at 932.5 eV belongs to Cu_2_O and other peak at 934 belongs to Cu which is in well agreement with XRD results of vacuumed anneal samples. To further elaborate Cu2p spectra, two peaks located at 934 and 952.4 eV were assigned to Cu2p_3/2_ and Cu2p_1/2_ respectively.

**Figure 8:**
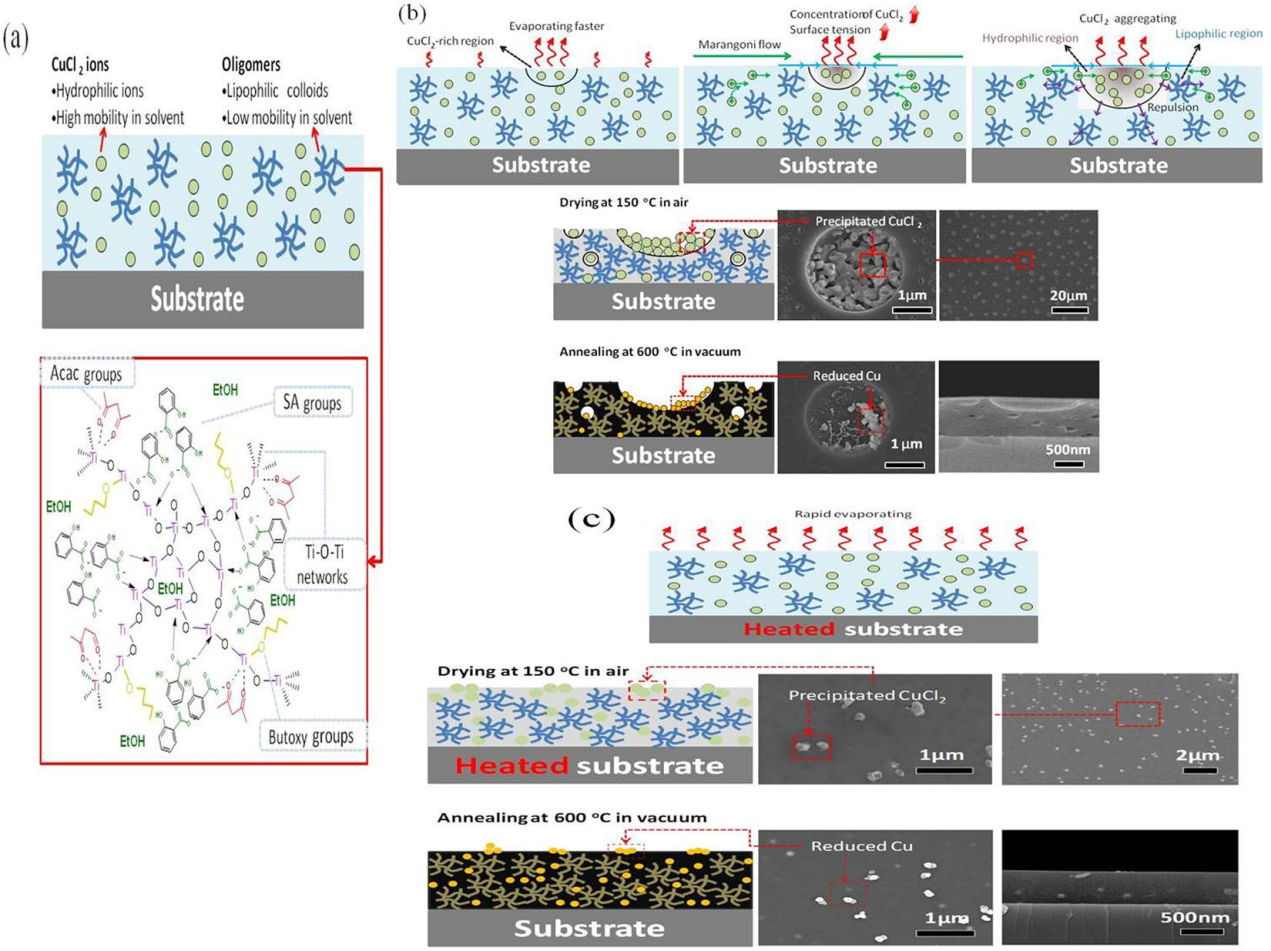
Illustration diagrams of the preparation mechanism. (**a**) Gel film on the substrate (**b**) normal solvent evaporation causing Marangoni flow results porous morphology (**c**) rapid evaporation suppressing Marangoni flow results nearly smooth surface.

On the basis of all above results a model of the preparation mechanism is established and elaborated in Fig. 8. The Fig. 8a shows the binary mixture in the gel film: the one is a Cu rich phase which is hydrophilic with the high mobility in solvent, and the other is a Cu poor phase, which are oligomers constituted of Ti-O network and other organic groups, such as Acac, SA group, and Butoxy group. These oligomers are lipophilic colloids with low mobility in solvents. The solvent of Cu rich phase, *i.e*. ethanol, evaporates faster, causing a surface tension at the interface which results the Marangoni flow from its surrounding area^27^. Then the CuCl_2_ gradually aggregates near the surface. From Fig. 8b, one can clearly see that the porous surface occurs with a precipitated CuCl_2_ inside the pores. This concludes that porous surface is created in gel-film stage, and after annealing at 600 °C, the precipitated CuCl_2_ is reduced to Cu but the porous surface is fixed during annealing process.

If the substrate is heated, the evaporation of the solvent in the Cu rich region is too fast to cause Marangoni flow, as shown in Fig. 8c, the surface tension will decrease and the Marangoni effect is suppressed. Therefore, nearly smooth surface was obtained except some white dots which are assigned to precipitated CuCl_2_ which reduced to Cu after annealing in vacuum at 600 °C. It’s concluded that in this mechanism, porous surface can’t be produce without Marangoni effect and the results of different surface morphologies due to this effect is shown in S1.

Based on such surface morphology, we believe that it can be used in many applications, for instance we provide the optical-thermal properties for the characterization as solar selective absorber (SSA). As the textured film materials have desired optical absorptive properties since it will generate light-trapping effect on the film surface which is beneficial for solar absorption. In the present work, we have shown that the composites of the sample prepared at various annealing temperature are quite different. As the UV-Vis diffuse absorption is not only effected by surface morphology but also highly dependent on the composite of the samples so it is hard to use these results (not shown here) to notify the enhancement of the absorptance. Actually, the enhanced absorption of the film by the porous surface has been discussed in detail in our previous work (ref. 24).

**Figure 9:**
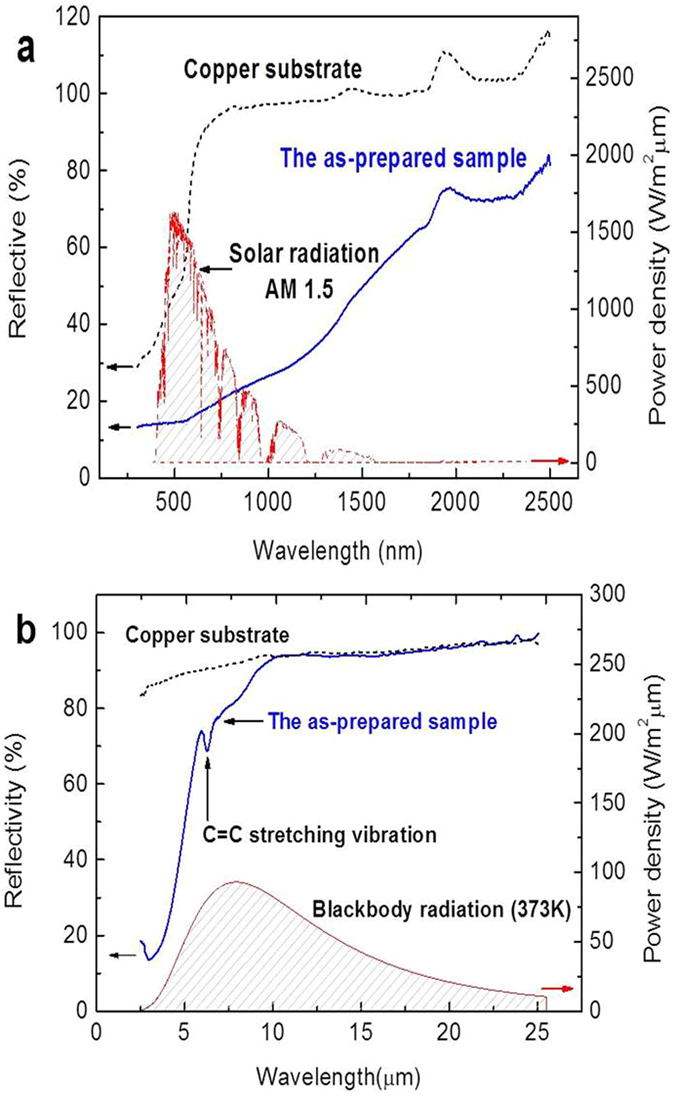
Optical performance (**a**) The reflectivity of samples in the range from 300 nm to 2500 nm (**b**) in the range from 2.5 μm to 25 μm.

The SSA is highly dependent on two optical parameters *i.e*. high absorptivity and low emissivity which is presented in following equations.

1 $$\alpha =\frac{{\int }_{0.3\mu m}^{2.5\mu m}{I}_{sun}({\rm{\lambda }})(1-{\rm{R}}({\rm{\lambda }})){\rm{d}}{\rm{\lambda }}}{{\int }_{0.3\mu m}^{2.5\mu m}{I}_{sun}({\rm{\lambda }}){\rm{d}}{\rm{\lambda }}}$$

2 $$\varepsilon =\frac{{\int }_{2.5\mu m}^{25\mu m}{I}_{B}({\rm{\lambda }})(1-{\rm{R}}({\rm{\lambda }})){\rm{d}}{\rm{\lambda }}}{{\int }_{2.5\mu m}^{25\mu m}{I}_{B}({\rm{\lambda }}){\rm{d}}{\rm{\lambda }}}$$

where *I*_*s*_ is the intensity of solar radiation (AM 1.5, ASTM G173-03, ISO), R represents the reflectivity of the sample, *I*_*B*_ is the radiation associated with blackbody at a given temperature. The reflectance spectra of Cu substrate, as prepared sample and solar irradiation in the range between 300–2500 nm are shown in Fig. 9a. The total reflectivity of Cu substrate is very high, but it decreases drastically for as-prepared sample especially in the solar irradiation dominating range, which demonstrates the significance of solar energy harvesting abilities. The reflectance in the range 2.5 to 25 µm is depicted in Fig. 9b. This figure also shows the reflectivity of Cu substrate and as-prepared sample. One can observe a small peak at 6.26 µm which could be assigned to C=C stretching vibration. The absorptance α and emittance ε are calculated using Eqs 1 and 2 and the resulting values are 0.76 and 0.18 respectively. The ε of the sample is slightly high which is an obstacle in practical application, since the emissivity contribution from presently used Cu substrate is high about 0.081 but the emissivity of film can be optimized by using better substrate or extra polished substrate.

## Conclusion

Sol-gel one step facile method on the basis of Marangoni effect has been established to fabricate a multi-scaled and controllable macro-porous structure films. In this proposed mechanism, the binary mixture of hydrophilic CuCl_2_ and lipophilic Ti-O network is used in sol to produce phase separation. The solvent of Cu rich phase, *i.e*. ethanol, evaporates faster, causing a surface tension at the interface which results the Marangoni flow from its surrounding area. A suitable evaporation rate in the gel film process leads to the macro-porous film. During the annealing process the macro-porous morphology of film sustained, which suggest that porous surface morphology has been created in gel film stage rather than due to annealing process.

The optical performance characterization of the composite film confirms an absorptivity of about 0.76 and emissivity of 0.17 without anti-reflective layer. Such values are slightly lower as compared to an ideal SSA but in this work we have hypothetically studied marangoni effect with temperature gradient and verified it, which is not used for any SSA before. Therefore, further investigation is needed in this area to enhance the optical properties of SSA.

## Experiment and Characterization

A facile approach to fabricate a porous surface is on the basis of hydrophilic-lipophilic balance (HLB) theory and the Marangoni effect using the sol-gel method. The Marangoni flow, a result of the dynamics of volatile binary liquid mixtures subjected to temperature or solute concentration driven surface tension gradient^28^, plays a key role in the application to obtain the textured morphology of surface coating^29–32^.

The film was prepared on quartz (40 mm × 40 mm × 1 mm) and copper (35 mm × 35 mm × 1.5 mm) substrates. The former is used for destructive characterization and the later is for performance characterization. The solution prepared by using mixture of tetrabutyl titanate (TBT), acetyl acetone (Acac), salicylic acid (SA), ethanol and copper chloride dehydrate (CuCl_2_·2H_2_O) with 1:2:2:50:0.8 molar ratios respectively. All reagents used in our analysis have been purchased from Sinopharm Chemical Reagent Co., Ltd and are used without further purification.

The sol was prepared using lipophilic TBT and hydrophilic CuCl_2._ Due to dissimilar nature of these compounds (HLB) results the phase separation. After about 48 hours of aging process (≤50% RH, at room temperature), the hydrolysis and poly-condensation of the precursor TBT was developed to a stable status. Due to the passivating effect of complexing agent SA the Ti-O-Ti network appeared and developed slowly but the other hydrophilic compound CuCl_2_ was still believed to be ionic in ethanol sol, which may be due to the fact that poly-condensation of Cu^2+^ in ethanol is hard to achieve and no catalyst were utilized to stimulate the process.

The spin coating of sol on substrate is performed by two ways to study Marangoni effect. At first, sol was spread on substrate at room temperature and secondly, the sol was spread on heated substrate. These two strategies have been used to analyze the Marangoni flow due to surface tension gradient, which is highly dependent on temperature and concentration changes. In the end, the prepared samples were annealed in two different atmospheres *i.e*. in Air, and Vacuum with different temperature ranges (150, 300, 450 and 600 °C). The sole purpose of annealing in vacuum was to obtain carbonization. The pyrolysis of SA leads to the formation of carbon more efficiently. Meanwhile, the reduction of Cu compounds by carbon is supposed to take place during the annealing process, while Ti element transform to titanium oxide instead of reducing it to metal by carbon, which is due to the fact that Ti has fairly low electro negativity^33^.

Thermogravimetry differential thermal analysis (TG-DTA) of xerogel was performed by thermal analyzer (Thermo plus TG 8210, Rigaku) with a heating rate of 5 °C/min from room temperature to 900 °C. For structural features of the films high-resolution transmission electron microscope (HRTEM, TecnailF20, FEI) at an accelerating voltage of 200 kV and scanning electron microscope (SEM, S-4800, Hitachi, at an accelerating voltage of 5 kV) were used. The film materials were worn out of the substrate for the preparation of samples for TEM analysis, and then ultra-sonically dispersed into ethyl alcohol for 20 min. The prepared solution was dropped onto the Cu grid and dried under an infrared lamp before TEM analysis. The phase analysis of composite film was carried out by X-ray diffraction (XRD, D/max 2550, Rigaku). Raman spectra was obtained using a Raman spectrometer (InVia, Renishaw) combined with 532 nm line of a laser as excitation with maximum laser power available *i.e*. 50 mW. UV-NIR spectrophotometer (Carry 500, Agilent) equipped with an integrating sphere using BaSO_4_ as a reference, at an incident angle of 3° 28′, in the range of (300–2500) nm was used. The chemical state of composite film was analyzed by X-ray Photoelectron Spectroscopy (XPS, KRATOS, AXIS, ULTRAD). Additionally, the reflectance from 2.5 to 25 μm was measured using a Fourier transform infrared spectrometer (FTIR, Tensor27, Bruker) for the optical performance characterization of the prepared composite.

## Electronic supplementary material

Supplementry information
