# Supplementary material for: Marangoni effect induced macro porous surface films prepared through a facile sol-gel route
Source: Sci Rep. 2017 Jul 13;7:5292. doi: 10.1038/s41598-017-05506-7 (PMC5509655; doi:10.1038/s41598-017-05506-7)
Supplement: Supplementary file 1 — Supplementry information [file 41598_2017_5506_MOESM1_ESM.pdf]

Supporting information

Marangoni effect induced macro porous surface films prepared through a facile sol-gel route

Shahid Khan[1], Kangkai Wang[1], Guangzhong Yuan[1], Mahmood ul haq[1], Zhizheng Wu[1], Muhammad Usman [3], Chenlu Song[1,2], Gaorong Han[1,2**], Yong Liu[1,2]*

1. State Key Laboratory of Silicon Materials and School of Materials Science and Engineering, Zhejiang University, Hangzhou 310027, China

2. Key Laboratory of Advanced Materials and Applications for Batteries of Zhejiang Province

3. Experimental Physics Laboratories, National Centre for Physics, Shahdara Valley Road, Quaid-i-Azam University, Islamabad, Pakistan

***Corresponding author: Yong Liu**

****Co-corresponding author: Gaorong Han**

State Key Laboratory of Silicon Materials,

School of Materials Science and Engineering,

Zhejiang University,

Hangzhou 310027,

P. R. Chin

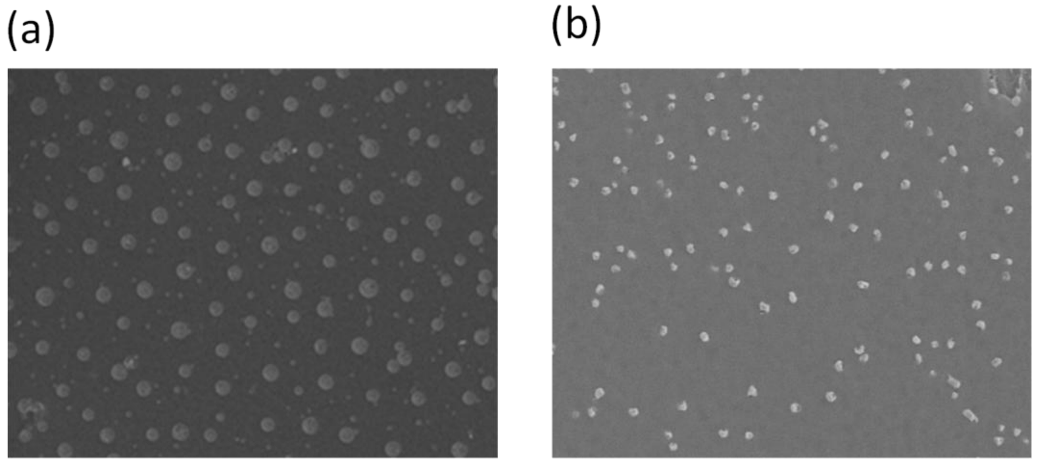

Figure1. Results of different surface morphologies (a) Due to Marangoni effect (b) without Marangoni effect
